# Functionalized Graphene Oxide with Chitosan for Protein Nanocarriers to Protect against Enzymatic Cleavage and Retain Collagenase Activity

**DOI:** 10.1038/srep42258

**Published:** 2017-02-10

**Authors:** Fatemeh Emadi, Abbas Amini, Ahmad Gholami, Younes Ghasemi

**Affiliations:** 1 Pharmaceutical Sciences Research Center, Shiraz University of Medical Sciences, Shiraz, Iran; 2 Department of Pharmaceutical Biotechnology, School of Pharmacy, Shiraz University of Medical Sciences, Shiraz PO Box 71468-64685, Iran; 3 Center for Infrastructure Engineering, Western Sydney University, Locked Bag 1797, NSW 2751, Australia

## Abstract

Proteins have short half-life because of enzymatic cleavage. Here, a new protein nanocarrier made of graphene oxide (GO) + Chitosan (CS) is proposed to successfully prevent proteolysis in protein and simultaneously retain its activity. Bovine serum albumin (BSA) and collagenase were loaded on GO and GO-CS to explore the stability and activity of proteins. SEM, AFM, TEM, DSC, UV-Vis, FT-IR, RBS, Raman, SDS-PAGE and zymography were utilized as characterization techniques. The protecting role of GO and GO-CS against enzymatic cleavage was probed by protease digestion analysis on BSA, where the protease solution was introduced to GO-BSA and GO-CS-BSA at 37 °C for 0.5-1-3-6 hours. Characterizations showed the successful synthesis of few layers of GO and the coverage by CS. According to gelatin zymographic analysis, the loaded collagenase on GO and GO-CS lysed the gelatin and created non-staining bands which confirmed the activity of loaded collagenase. SDS-PAGE analysis revealed no significant change in the intact protein in the GO-BSA and GO-CS-BSA solution after 30-minute and 1-hour exposure to protease; however, free BSA was completely digested after 1 hour. After 6 hours, intact proteins were detected in GO-BSA and GO-CS-BSA solutions, while no intact protein was detected in the free BSA solution.

Nanocarriers for protein delivery have attracted remarkable attention with great potentials in advanced medical therapy^1^. With recent nanobiotechnology progress in medicine and pharmaceutics, a broad range of nanocarriers with diverse sizes and surface properties have been designed to allow systemic (intravenous and oral) or local (mucosal) administration^2^,^3^,^4^. Compared with other small-molecule drugs, proteins have several advantages, such as a higher affinity and specificity, less adverse effects, and greater effectiveness in the treatment of genetic or refractory diseases^5^. Despite these advantages, protein therapeutic development has had a slow progress due to proteins’ rapid kidney clearance and susceptibility to cleaving by proteolytic enzymes. This results in their short half-life and low stability in biological environments^5^,^6^. Thus, to overcome the instability of proteins against enzymatic cleavage^7^,^8^,^9^, many approaches have been proposed, such as pegylation, liposomal formulations, glycosylation, transferrin fusion, etc.^10^,^11^,^12^. Despite these approaches, the stability of proteins against proteolysis in biological environment has remained a challenge for researchers and, thus, for clinical applications^13^,^14^.

In this study, graphene oxide (GO) was proposed to protect proteins against proteolysis. GO-based nanomaterials provides an effective platform for protein delivery^15^. GO, prepared from natural graphite, is a stable precursor for chemically converted graphene^16^. This nanomaterial has found widespread biomedical applications in drug/gene delivery^17^,^18^, enzyme stabilization^19^, photo-thermal therapy in cancer^20^,^21^,^22^, biological sensing and imaging usages^23^,^24^,^25^, antibacterial activity^26^,^27^,^28^, and as a biocompatible scaffold for cell culture in tissue engineering^29^,^30^,^31^. Authors have shown that the bio-applications of GO is originated from its high specific surface area, electronic conductivity, thermal conductivity, mechanical strength, and low cost of scalable production with facile biological/chemical functionalization^32^,^33^,^34^,^35^,^36^,^37^,^38^.

To improve the solubility, biocompatible molecules are attached to nanoparticles via a functionalization process^39^,^40^. Here, we propose grafting chitosan (CS) on GO sheets to efficiently improve their desired properties for protein delivery purposes^41^. CS is non-toxic and has a high biocompatibility and biodegradability, low immunogenicity, and antibacterial properties, as well as pH-responsivity and good solubility^42^,^43^. Thus, it can be a good pharmaceutical ingredient in drug carriers and gene delivery systems^44^.

In this work, GO was synthesized and attached to CS, then characterized by AFM, TEM, SEM, Raman, Rutherford Backscattering spectrometry (RBS) and FTIR. BSA (bovine serum albumin) as a model protein was loaded on GO and GO-CS to investigate its stability and protection against trypsin using SDS-page analysis. Also, collagenase was loaded on GO and GO-CS to probe its activity before/after loading using gel zymography analysis. Collagenase (matrix metalloproteinase, MMP) is one of the important therapeutic proteins in medical treatments for several diseases, including necrotic tissue, Dupuytren’s contracture and Peyronie^45^, chronic total occlusion^46^, lumbar disc herniation^47^, oral submucus fibrosis^48^, and tumors^49^,^50^.

It is found that GO and GO-CS can protect the protein against enzymatic cleavage, and the collagenase still retains its activity after loading on GO or GO-CS. Therefore, using GO-CS as a protein therapeutic nanocarrier can increase the half-life of proteins in biological environment, reduce the frequency of administration, lower drug doses, and optimize the costs related to protein therapy in particular for intravascular and oral administration of therapeutic proteins.

## Results and Discussion

### Characterizations of GO and GO-CS

**Figure 1:**
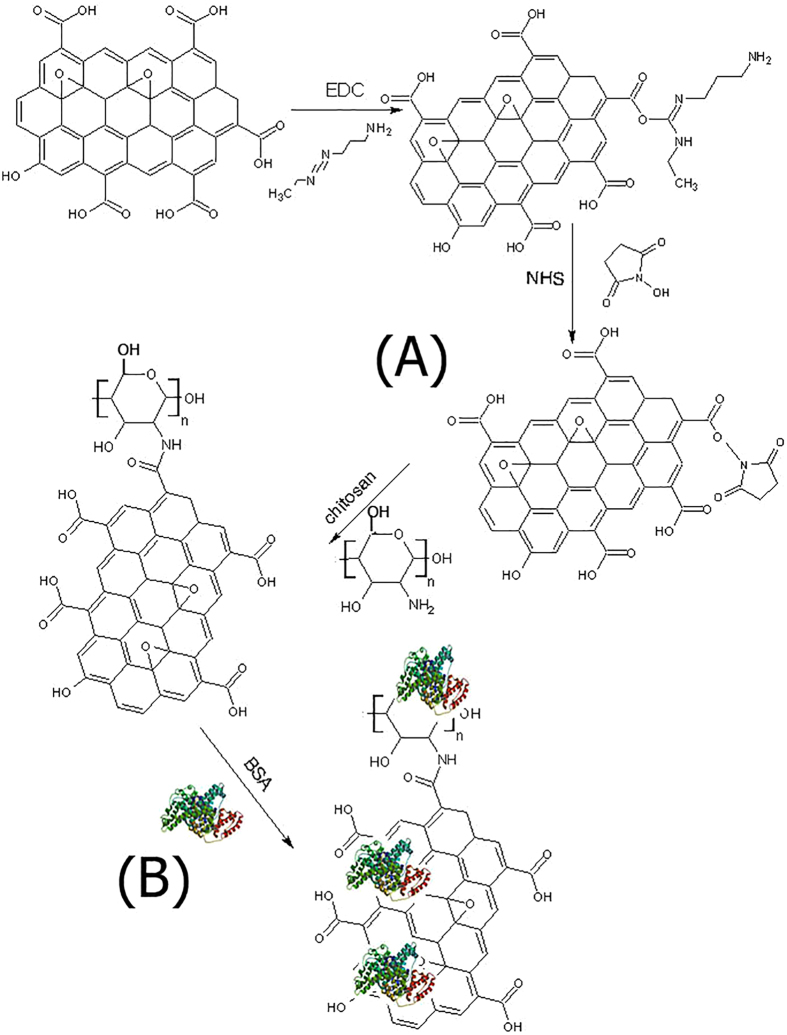
Schematic diagram of GO-CS formation through BSA loading on GO-CS.

**Figure 2:**
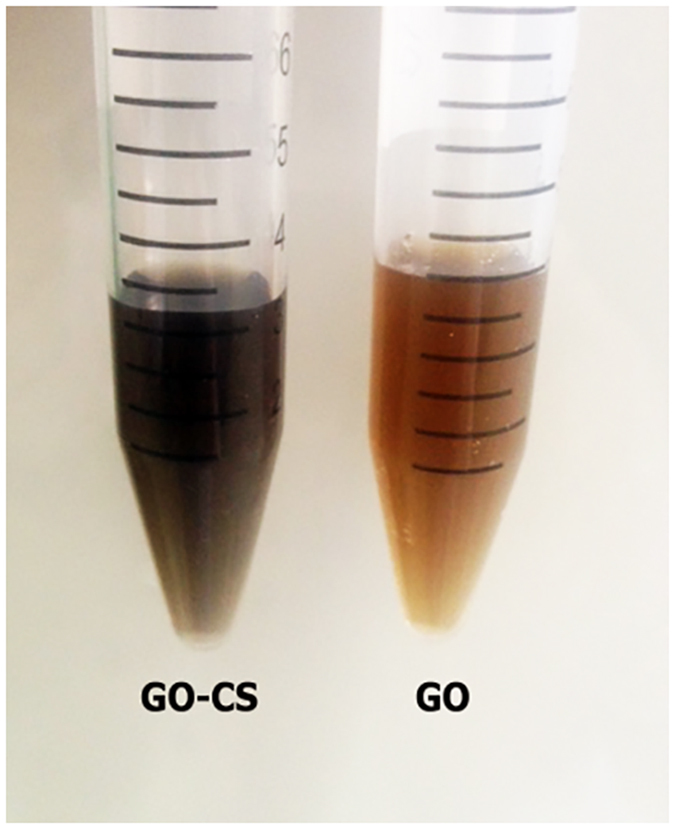
The appearance of GO before and after binding with CS at a concentration of 5 mg/ml.

**Figure 3:**
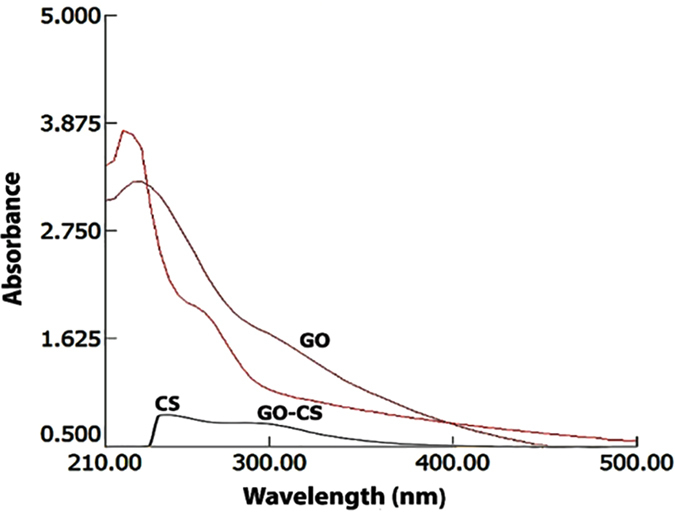
UV-visible spectra of GO, GO-CS and CS. The spectral range is 210–500 nm with the GO peak at 230 nm.

GO was synthetized by Hummer’s method^51^, in which the graphite was preoxidized by sulfuric acid, and the resultant preoxidized graphite was further oxidized with KMnO_4_ to achieve oxygen-containing groups (such as carboxylic acid, epoxy and hydroxyl) on graphite surface^52^. Extra potassium permanganate was used to increase oxidation^51^,^53^. GO was attached to a low molecular weight chitosan via amide linkage in the presence of N-(3-Dimethylaminopropyl)-N′-ethylcarbodiimide hydrochloride (EDC), N-Hydroxysuccinimide (NHS) in 2-(N-Morpholino)-ethanesulfonic acid and 4-Morpholineethanesulfonic acid (MES buffer). Figure 1A illustrates that CS attached to GO via amide linkage of GO carboxylic acid and CS amine groups in the presence of EDC and NHS in MES buffer. The carboxylic acid groups of GO were activated by EDC to initiate the formation of an active ester between GO and CS, which was stabilized further by NHS. The active ester reacted with the amine groups on CS, forming an amide bond between GO and CS^41^,^54^. An excess amount of CS was added to consume more carboxylic groups on GO, and the unreacted CS was eliminated by filtration and dialysis. Figure 2 shows the GO solution before and after CS coating procedure; the color of GO was yellowish brown before CS coating and became black after CS coating procedure. UV-Vis spectra were obtained from 200 to 500 nm for the GO, CS and GO-CS dispersed in water (Fig. 3). According to previous studies^51^,^53^,^55^,^56^,^57^,^58^, an intense peak at 230–235 nm wavelength is the characteristic peak of GO related to the *π* − π* transition of C=C, and a small shoulder at around 300 nm is related to the *n* − π* transition of C=O, and the CS peaks are at 240 nm and 280 nm. After attachment with CS, the peaks of GO at 230 and 300 nm shifted 20 nm to the left.

**Figure 4:**
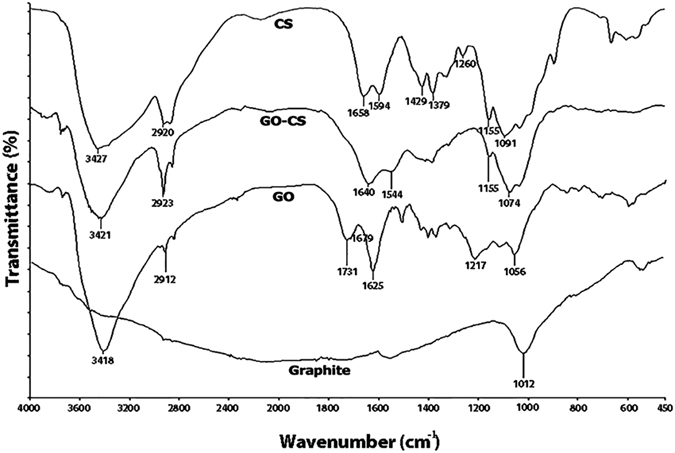
FTIR spectra of GO, GO-CS, graphite and chitosan for the wavelength range of 450–4000 cm^−1^. The disappearance wavelength at 1731 cm^−1^ and new wavelength appearance at 1640 cm^−1^ in the GO-CS spectrum prove the attachment of GO with CS.

The FT-IR analysis of GO in Fig. 4 shows the existence of H-bonded OH stretch at 3418 cm^−1^, C-H stretch at 2912 cm^−1^, C=O in carboxylic acid group at 1731 cm^−1^, C=C in conjugated ketones at 1625 cm^−1^ and 1679 cm^−1^, phenol C-O stretch at 1217 cm^−1^, and primary alcohol C-O stretch at 1056 cm^−1 ^59. The presence of oxygen-containing groups on GO is observed while graphite spectra have no significant peak for these groups^55^,^56^,^58^,^60^,^61^. Most of the absorbance peaks of CS and GO-CS are overlapped. The FT-IR spectrum of GO-CS contains the stretching vibrations of NHCO at 1640 cm^−1^. The NH_2_ groups of chitosan chains react with the carboxylic acid groups of GO to form an amide linkage^62^. Compared with GO, the disappeared peak of carboxylic acid at 1731 cm^−1^ is an evidence that the carboxylic groups reacted with the NH_2_ groups in CS to form an amide linkage (Fig. 1A)^52^,^55^. In the spectrum of GO-CS, the peak of primary alcohol C-O stretch at 1074 cm^−1^ was more intense than that of GO at 1056 cm^−1^, due to the interaction of OH groups of GO with CS^63^. The characteristic signal of the secondary amide (N–H bending) shifts from 1594 cm^−1^ in CS spectrum to 1544 cm^–1^ in GO-CS spectrum, which indicates the presence of newly formed amide bonds between GO and CS^41^. In short, the FT-IR results clearly confirmed the attachment of CS with GO.

**Figure 5:**
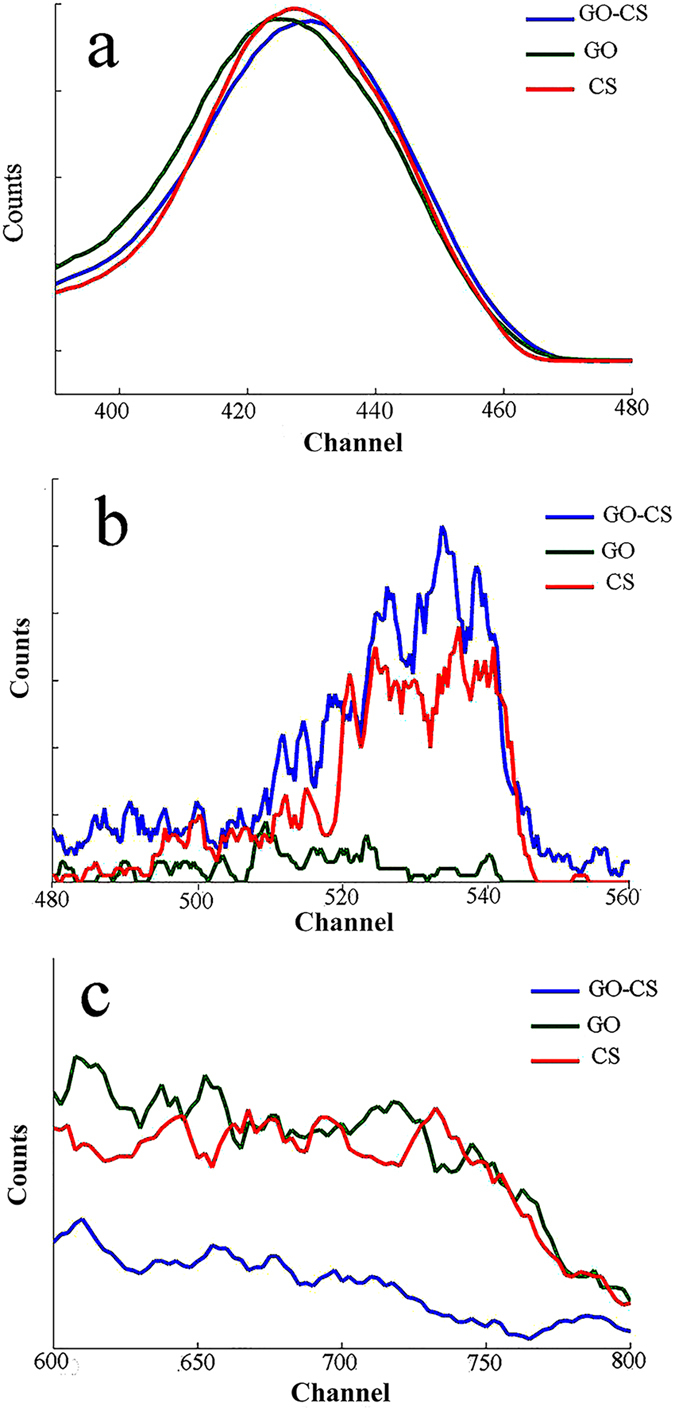
RBS analysis for detecting the elemental composition of GO, CS and GO-CS. Carbon region from channel 400 to 460 (**a**), nitrogen region from channel 500 to 540 (**b**), and oxygen region from channel 600 to 750 (**c**).

Figure 5 and Table 1 show the results from RBS analysis to detect the elemental composition of CS, GO and GO-CS; the regions related to carbon (400–460), nitrogen (500–540) and oxygen (600–750) were separated for clarity. The O/C ratio of GO and CS was 0.54 and 0.73, respectively, and after the attachment of GO with CS, the O/C ratio decreased to 0.47. The O content of GO, CS and GO-CS was 35%, 35% and 26%. These data indicated that the oxygen content and O/C ratio of GO decreased and resulted in GO reduction after attachment with CS. Carboxylic groups of GO reacted with NH_2_ groups of CS, and GO lost the oxygen from its carboxylic group to form amide linkage (Fig. 1A). The color of GO changed from yellow brown to black (Fig. 2)^56^.

**Figure 6:**
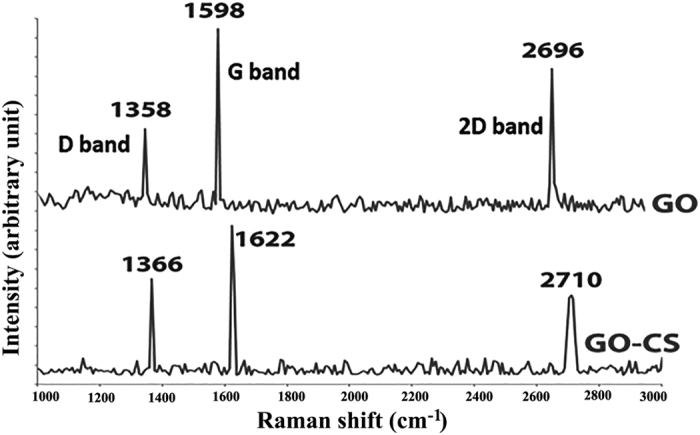
Raman spectrum of GO and GO-CS. 2D peak in GO-CS spectrum is broadened and shifted to upper 2700 that shows changes in the GO layers by the attachment to CS. The shifts in D, G and 2D bands and the change in intensity ratio of D/G revealed that GO was successfully functionalized by CS.

Raman analysis was employed to study the layers of GO and the attachment of GO with CS. The characteristic peaks of Raman spectrum of GO are D, G and 2D bands located at 1358, 1595 and 2696 cm^−1^, respectively. The G and 2D bands of single-layer GO sheets typically are detected at 1585 and 2679 cm^−1^. The positions of G and 2D bands of multi-layer GO sheets (2–6 layers) shift to lower and higher wavenumbers by 6 and 19 cm^−1 ^64. Here, G and 2D band shifted to higher wavenumbers by 13 and 17 cm^−1^ which confirmed the synthesis of multilayer GO sheets. According to Fig. 6, the intensity ratios of D/G and 2D/G are 0.53 and 0.81, respectively, which are corresponded to bilayer GO^64^. Therefore, bilayer or multilayer GO sheets were successfully synthesized. In addition, in GO-CS spectrum, D and G bands shifted to higher wavelengths that related to disturbance of GO surface by additional chemical bonds between carbon groups of GO and functional groups of CS. The intensity ratio of D/G shows the stacking behavior of GO, increased to 0.65, indicating the attachment of GO with CS^65^. 2D band is very sensitive to changes in the number of GO layers^66^. In the GO-CS spectrum, 2D is broadened and shifted to upper 2700 cm^−1^ that shows changes in the GO layers after attaching to CS. The D, G and 2D bands shifts and the changes in the intensity ratio of D/G revealed that GO was successfully functionalized by CS.

**Figure 7:**
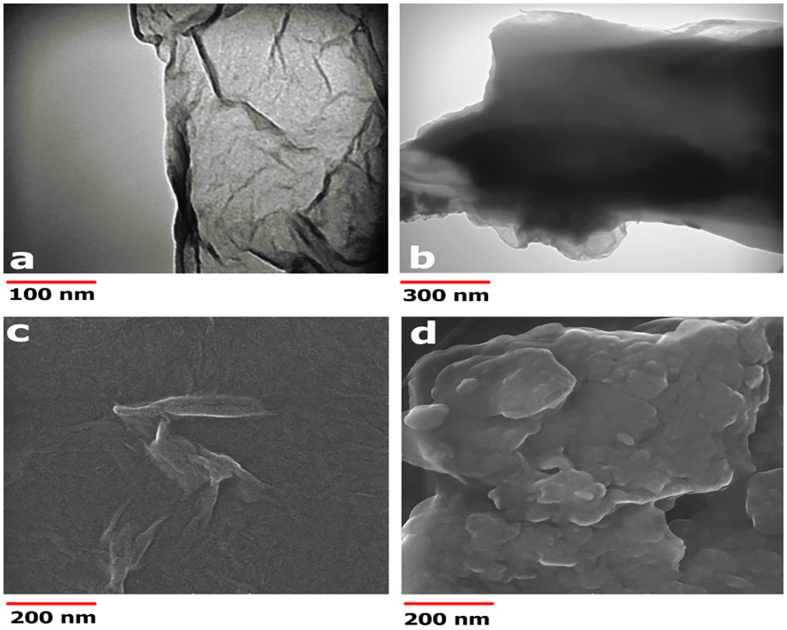
TEM images, on formvar-carbon coated copper grids of: (**a**) GO with a wrinkled surface and thin sheet-like structure, and (**b**) denser and thicker GO-CS. The scale bars are 100 nm for GO and 300 nm for GO-CS. SEM images of: (**c**) GO with a smooth surface, and (**d**) GO-CS with a rough surface. The scale bar represents 200 nm for the view field of 1.38 μm.

The morphology of GO and GO-CS was characterized by TEM, SEM and AFM techniques. A TEM micrograph of GO nano-sheets is shown in Fig. 7a. GO is an extended thin sheet with a wrinkled surface^56^,^61^,^67^,^68^,^69^. It has been shown that this wrinkled nature is an advantage as it provides a larger surface area to prevent a collapse back to a graphitic structure^56^. The TEM image of GO-CS shows that chitosan covered the GO sheets and thickened the virgin GO (Fig. 7b). The observed morphology of GO-CS can be attributed to the amide bond between amino groups of the CS and carboxyl groups of GO, as well as the hydrogen bonds between hydroxyl and amino groups from the CS and oxygen groups of GO (Fig. 1A)^56^. Compared with GO-CS, the larger and smoother surface of GO was confirmed by the SEM micrographs in Fig. 7c^70^,^71^,^72^,^73^. The attachment of CS to GO resulted in obvious changes in surface morphology and roughness of virgin GO (Fig. 7d)^71^,^74^.

**Figure 8:**
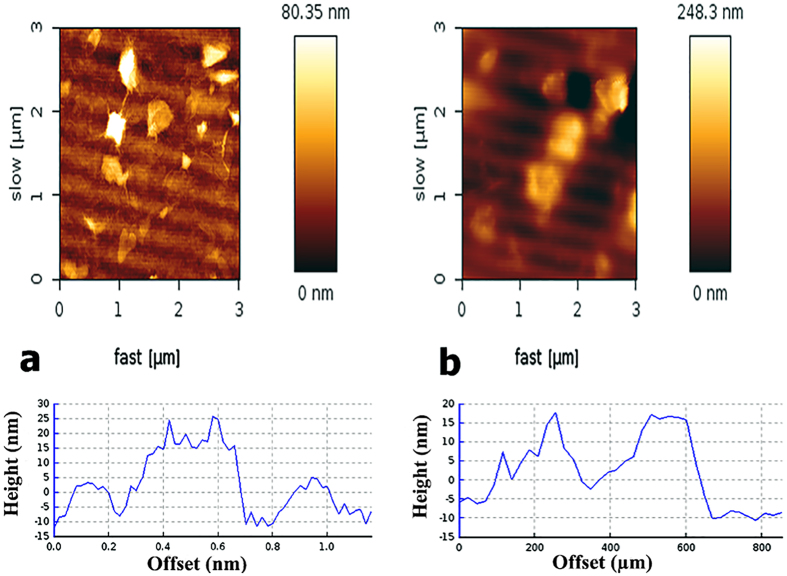
Contact mode AFM images and height profile of: (**a**) GO with sharp edges and flat surfaces; the thickness range is 0.6–10 nm, and (**b**) GO-CS with coarse edges and protrusions on its surface; the thickness range is 10–25 nm.

**Figure 9:**
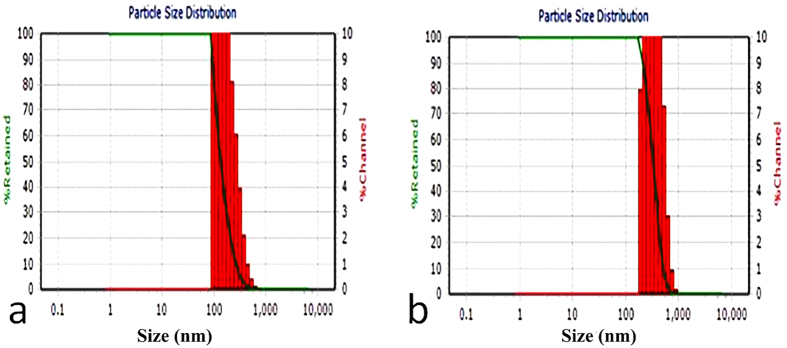
DLS analysis of (**a**) GO with an average size 150 nm, and (**b**) GO-CS with an average size of 350 nm.

The observed thicknesses of GO and GO–CS sheets were characterized using the AFM method. As shown in Fig. 8a, GO sheets have sharp edges^41^,^51^,^53^,^55^,^56^; in contrast, the GO-CS sheets have coarse edges with some protrusions on their surface (Fig. 8b)^41^,^56^. The thickness of GO sheets is in the range 0.6–10 nm which demonstrates the exfoliation of GO sheets to few layers sheets. The variation in GO thickness is due to the stacking of GO layers together during the sample preparation for AFM examination^75^. The topographic height of GO-CS increased to 10–25 nm, indicating the successful grafting of CS onto the surface of GO. The variation in thickness is correlated to the bundles of CS chains attached to the GO sheets^56^. According to the DLS (Dynamic Light Scattering) results in Fig. 9, GO average size is 150 nm and after attaching to CS, the GO-CS average size increases to 350 nm.

### Evaluation of BSA loading efficiency

**Figure 10:**
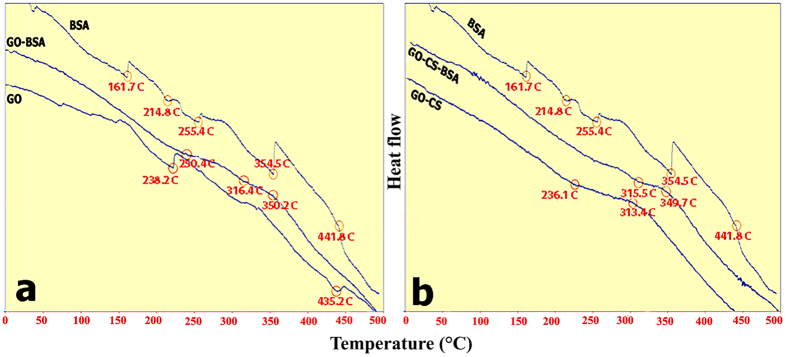
DCS spectra of GO, GO-CS, BSA, GO-BSA, and GO-CS-BSA at a heating rate of 10 °C min^−1^ from 40 to 500 °C.

**Figure 11:**
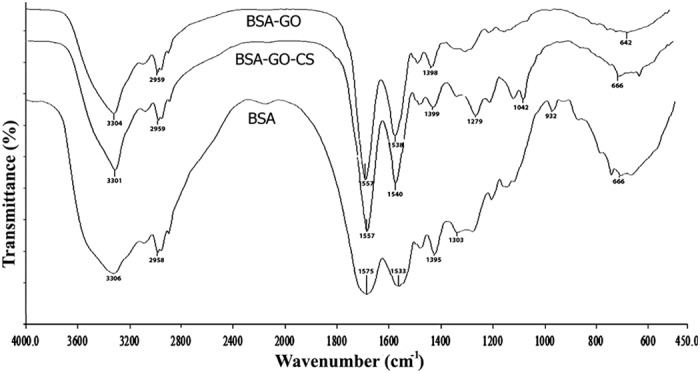
FTIR analysis of BSA before and after loading on GO and GO-CS. The peak of oxygen-containing groups of GO was disappeared after loading of BSA on GO.

Bradford reagent forms a complex between Coomassie Brilliant Blue and proteins. As a result, a protein-coomassie blue complex is created, which is evidenced by the absorbance maximum at 595 nm^76^. The concentrations of free BSA on the supernatant of GO and GO-CS were 0.74 mg/ml and 0.37 mg/ml, respectively. When the protein was loaded on GO, the loading efficacy (LE) was 26% and after loading the protein on GO-CS, it increased to 63%. In addition, Differential scanning calorimetry (DSC) analysis explained the difference in stability between naked GO, GO-CS, GO-BSA, GO-CS-BSA and BSA. DSC diagrams (Fig. 10) indicate that the functional groups of GO interacted with CS and BSA, increased the thermal resistance with a higher stability for BSA. FTIR analysis in Figs 4 and 11 revealed that, after loading BSA on GO, the peak intensity of GO at 1731 cm^−1^ (carboxylic acid), 1217 cm^−1^ (phenol) and 1056 cm^−1^ (primary alcohol) either decreased or disappeared implying the reduction in GO^77^. Figure 1B shows the loading of BSA, where the protein is loaded on GO-CS based on physical immobilization via *π* − *π* stacking and other molecular interactions. The physical loading on GO occurs via different types of interactions, such as Van der Waals forces, electrostatic or hydrophobic π − π stacking interactions and hydrogen bonds between the oxygen functional groups of GO and nitrogen/oxygen groups of protein^78^,^79^. Similarly, the interaction between protein and CS is on the basis of Van der Waals forces, electrostatic and hydrophobic interactions, and hydrogen bonds^79^. Compared with GO alone, LE is increased in GO-CS, while loading BSA on GO-CS improves the biocompatibility and solubility of GO^41^,^56^. It seems that non-covalent loading of protein on nanostructures has some benefits compared to covalent binding, as covalent immobilization may cause steric modifications in the protein, decreasing the protein’s functionality and activity^79^.

### Stability against enzymatic cleavage

**Figure 12:**
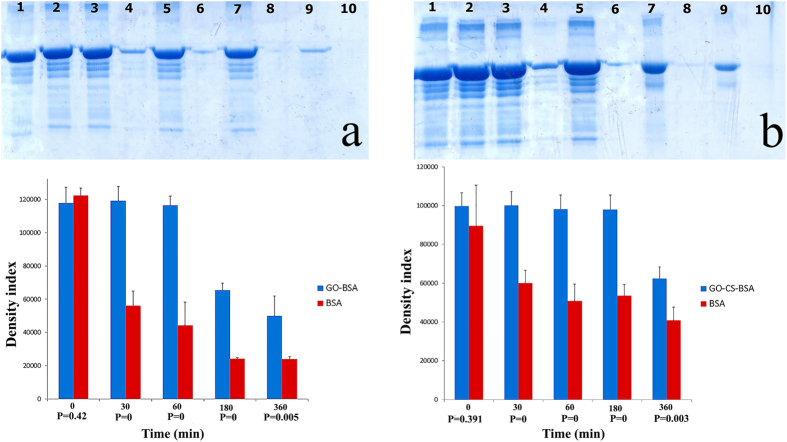
Statistical data and p-values of: (**a**) SDS analysis of GO-BSA, free BSA (as control) before adding trypsin (bands 1, 2), GO-BSA and free BSA 30 minutes after adding trypsin (bands 3, 4), 1 hour (bands 5, 6), 3 hours (bands 7, 8), and 6 hours (bands 9, 10); (**b**) SDS analysis of GO-CS-BSA, free BSA (as control) before adding trypsin (bands 1, 2), GO-CS-BSA and free BSA 30 minutes after adding trypsin (bands 3, 4), 1 hour (bands 5, 6), 3 hours (bands 7, 8), and 6 hours (bands 9, 10).

SDS-PAGE gels, statistical data and the p-values are shown in Fig. 12(a,b) to determine the significant differences between free BSA and loaded BSA on GO or GO-CS after adding trypsin at the specified time intervals. After 30 minutes, 1, 3 and 6 hours, GO-BSA solution contained intact BSA and GO could protect BSA against trypsin (bands 3, 5, 7 and 9 in Fig. 12a) as well as in the GO-CS-BSA samples (bands 3, 5, 7 and 9 in Fig. 12b). Free BSA (band 4, 6, 8 and 10 in Fig. 12a,b) was significantly digested at the same time intervals (p-value ≤ 0.01). The amount of both free protein and loaded protein decreased gradually, but the speed of enzymatic digestion of loaded protein was even slower. These results suggest that the loaded BSA on GO and GO-CS was significantly (P-value ≤ 0.01) protected against trypsin at each time interval. Similar results of the effects of GO-PEG against enzymatic cleavage were reported by Shen *et al*.^80^ Although the mechanism of GO and GO-CS for protecting BSA against enzymatic digestion is still unclear, it has been shown that the steric hindrance of GO has a key role in preventing trypsin from binding with protein to digest^80^,^81^. Another probable mechanism is the reductant effect of BSA on GO^77^. According to Figs 4 and 11, the oxygenated groups of GO were disappeared after BSA loading on GO. The reduced GO aggregates and wraps around trypsin and makes a barrier between trypsin and BSA, which in turn increases the digestion time.

### Collagenase loading on GO and GO-CS

According to the standard curve from Bradford assay, the LEs of collagenase were 35% and 72% for GO and GO-CS, respectively. The loading difference between BSA and collagenase is attributed to the different functional groups on the surface, charges and isoelectric point of the specified proteins and nanoparticles^82^. In the protein-nanoparticle conjugates, many variables, such as the chemical structural features, surface charge and properties of both nanoparticle and protein, affect the loading efficiency^83^. By comparing the values of zeta potential of BSA and collagenase, loaded on GO and GO-CS, from Tables 2 and 3 it is found that protein loading enhances via electrostatic interaction between proteins and positive nano-carriers. In fact, the LE value is increased from 26% for GO with negative zeta potential to 63% for GO-CS with positive zeta potential.

### Zymography analysis

**Figure 13:**
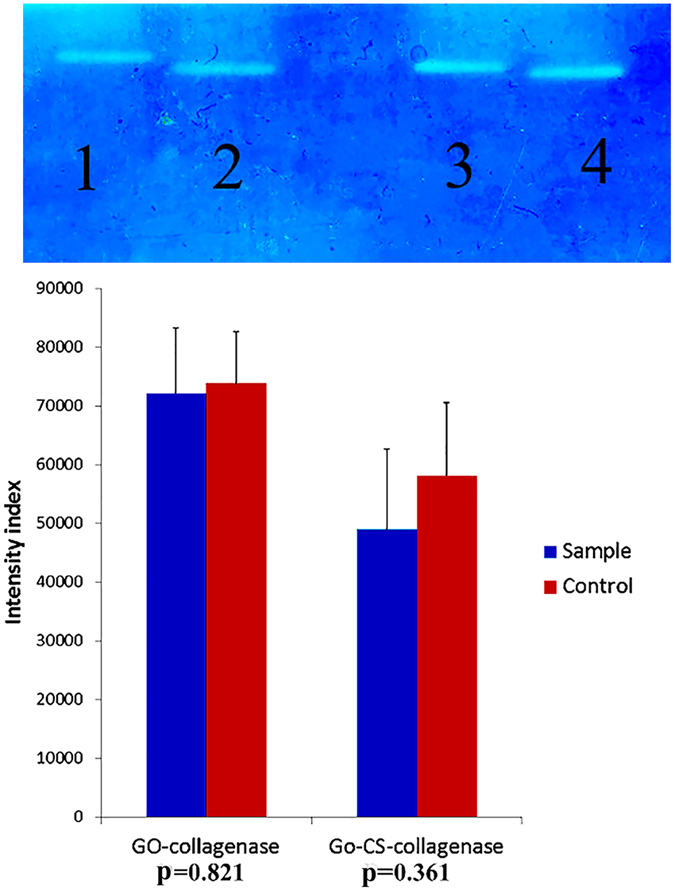
Statistical data and p-values of zymographic analysis: (bands 1, 2) for GO-collagenase, free collagenase (as control); (bands 3, 4) for GO-CS-collagenase and free collagenase (as control).

The non-staining bands in the gel zymogram show the activity of collagenase; and, the lower density indicates the higher collagenase activity (Fig. 13). Due to the different LEs of collagenase on GO and GO-CS, each one has its control at the same concentration of collagenase without nanoparticle. Although both GO and GO-CS enhance the collagenase activity (lower density in Fig. 13), there is no significant activity difference between loaded and free collagenase (p-values 0.361 and 0.821).

To sum up briefly, graphene oxide was functionalized with chitosan to produce a new GO-CS nano-carrier for proteins to protect them against proteolysis, extend their half-life and retain their activity and stability in biological contexts. The morphological analysis by AFM confirms there were a few layers of GO with a total thickness of 0.6–10 nm, which increased after the functionalization with CS to 10–25 nm. The characteristic peaks for GO and GO-CS were 230 and 210 nm, respectively, confirming the successful attachment of GO to CS. Bovine serum albumin, as a model protein, was loaded on both GO and GO-CS to investigate the loading efficacy and stability against trypsin. The loading efficacy of BSA loaded GO rose from 26% to 63% when it was loaded on GO-CS, along with an improvement in the thermal resistance of BSA after loading on both GO and GO-CS. A comparison between the protection role of GO and GO-CS against enzymatic cleavage was carried out by protease digestion analysis on BSA of GO-BSA and GO-CS-BSA, by introducing a trypsin solution at 37 °C for 30 minutes, 1, 3 and 6 hours. SDS-PAGE analysis revealed no significant change in the intact protein in the GO-BSA and GO-CS-BSA solutions after 30 minutes and 1 hour exposure to trypsin. However, free BSA was completely digested after 1 hour, while intact proteins remained in the GO-BSA and GO-CS-BSA solutions even after 6 hours. According to SDS page observation, GO and GO-CS protected the protein against enzymatic cleavage. In fact, the advantage of GO-CS over toxic GO, is its higher efficacy of BSA loading on GO-CS which makes it more biocompatible^84^. It is also revealed that the collagenase remains active after loading on GO and GO-CS. In short, due to the protection effect against trypsin with higher protein stability and activity, GO-CS can be a new nano-carrier for protein delivery via different routes of administration such as intravenous infusion and oral applications. Here, we just demonstrated the protection effect of GO-CS against trypsin; however, due to the variable effects of GO on enzymes, further work is required to investigate its effect on other proteases with considering all variables.

## Materials and Method

GO was prepared according to Hummer’s method in 5 steps with some modification^51^: (1) 1 g of graphite powder was dissolved in 23 ml of 98% H_2_SO_4_ (v/v) and stirred for 3 days in a three-neck flask. (2) The product was placed in an ice-water bath and 6 g of KMnO_4_ was gradually added to the solution; the color changed from black to dark green. (3) It was moved to an oil bath at 40 °C and stirred for 30 minutes, then heated at 70 °C for 45 minutes; after heating, the color of the mixture changed to dark brown. (4) 6 ml of distilled water (DW) was added to the three-neck flask and heated at 105 °C for 10 minutes. Subsequently, another 40 ml of DW was added to the suspension with the temperature maintained at 100 °C for 15 minutes. The reaction was terminated by adding 150 ml of DW and 15 ml of H_2_O_2_ 35% (v/v) solution. At the end of this stage, the color changed to yellowish brown. (5) Finally, GO was washed by centrifugation at 10,000 g for 5 minutes. The supernatant was discarded and the precipitate was washed two times with 5% HCl (v/v) and five times with DW.

GO-CS was prepared by the amidation of GO with CS in the presence of EDC and NHS^51^. CS (0.125 g) and GO (0.1 g) were dispersed in 25 ml of MES buffer (0.1 M, pH adjusted to 6) in a bath sonicator at room temperature for 1 hour to obtain a homogeneous colloidal suspension. Then, EDC (0.3 g) and NHS (0.4 g) were gradually added to the flask under nitrogen gas within 20 minutes. The suspension was again sonicated at room temperature underwent bath sonication for 5 hours, and then stirred for 18 hours. The product was filtered over a 0.2 micron nylon microporous membrane and washed using a large amount of acetic acid solution (0.1 M) to remove unreacted CS. After that, the collected solid was re-dispersed and dialyzed (molecular weight cut-off 10 kDa) with DW for four days at room temperature. The CS loading on GO substrates was demonstrated using the software ChemSketch 12.00. An amide linkage was formed between GO carboxylic acid and CS amine groups in the presence of EDC and NHS within MES buffer (Fig. 1A).

TEM micrographs were obtained using a Zeiss EM900 (Carl Zeiss, Germany) microscope operating at 80 kV on formvar-carbon coated copper grids. AFM images were recorded in the contact mode using Nano wizard II (JPK, Germany). A Fourier transform infrared (FT-IR) spectrometer (Perkin Elmer, UK) was used by applying KBr pellet to record the FT-IR spectra. RBS was performed with 3 MeV electrostatic Van de Graaff accelerator (High voltage, USA). Raman spectroscopy was obtained at room temperature using Avaspec 3648 (Avantes, Netherland) with 532 nm diode laser. VEGA2-LMU microscope (Tescan, Czech) was used for SEM characterization. The UV-Vis spectra were recorded using a T80+ UV-Vis spectrometer (PG instruments, Australia). DSC analysis was performed using a DSC machine, BÄHR Thermoanalyse GmbH, Type 302 (Hüllhorst, Germany), while the temperature was raised from 40 to 500 °C at a rate of 10 °C min^−1^. Zeta potentials and DLS analysis was obtained by zetacheck and nanoflex (microtrac, Germany).

Protein loading on GO and GO-CS was achieved by overnight mixing BSA (1 mg/ml) with an aqueous solution of GO or GO-CS (1 mg/ml) in darkness at room temperature, and the unbound BSA was removed by centrifugation. The loading of BSA on GO and CS is depicted in Fig. 1B. The protein concentration in the supernatant was analyzed with a UV spectrophotometer at a wavelength of 595 nm using the Bradford protein assay. The protein loading was investigated using the DSC technique^76^.

Triplicate samples were used to analyze the protein loading efficiency (LE) using Eq. 1:


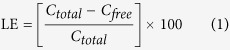


where *C*_*total*_is the total concentration of BSA in the solution, consisting of free and loaded BSA, and *C*_*free*_represents the concentration of free BSA that remained in the supernatant after centrifugation.

In each assay, the same concentration of protein in GO-BSA, GO-CS-BSA and free BSA was treated with trypsin aqueous solution (0.125%) at 37 °C. To investigate the intact proteins, the samples were analyzed by sodium dodecyl sulfate polyacrylamide gel electrophoresis (SDS-PAGE 10%) at four time intervals (30 minutes, 1, 3 and 6 hours). For each time interval, the samples were heated to 100 °C within 10 minutes to stop the reaction and then immediately analyzed by SDS-PAGE^80^.

Collagenase was used as a protein to investigate the effects of GO and GO-CS on therapeutic protein activity. First, collagenase was loaded on nanoparticles by mixing GO and GO-CS with collagenase. The unbound proteins were measured by the Bradford assay and the LE was calculated using Eq. 1. For loading the components into the wells of gel zymography, the GO-collagenase, GO-CS-collagenase and free collagenase were mixed in the same concentration with a zymogram sample buffer consisting of: Tris buffer pH 6.8 (0.5 M), SDS (10%), glycerol (20%) and bromophenol blue (0.05%). The resolving gel consisted of: 1.5 M Tris buffer pH 8.8, 10% (w/v) sodium dodecyl sulfate (SDS), 30% polyacryamide, 10% (w/v) ammonium persulfate (APS), tetramethylethylenediamine (TEMED), DW and 1% Gelatin. The stacking gel consisted of: 0.5 M Tris buffer pH 6.8, 10% SDS, 30% polyacrylamide, 10% (w/v) ammonium persulfate (APS), TEMED and DW. Electrophoresis was carried out at 35 mA for 3 hours; then, the gel was removed and incubated in a renaturation buffer (Triton X-100 2.5% in Tris HCl 50 mM) at room temperature. After 1 hour, the renaturation buffer was decanted and gel was incubated in the developing buffer (10 mM CaCl_2_, 0.2 M NaCl in Tris HCl 50 mM) for 18 hours at 37 °C. The resultant gel was stained with coomassie blue for 1 hour and destained with 10% acetic acid and 40% methanol. The areas that corresponded to collagenase activity were considered as non-staining bonds on the gel^85^,^86^. Image j software (1.44 P) was used to quantitatively determine the density of SDS-PAGE bands and gel zymography. Statistical analysis was performed using student’s t-test to determine p-values. Differences were considered to be significant when the p-value ≤ 0.01. Data (density of bands) were expressed as the mean ± SD (n = 4).

## Additional Information

**How to cite this article:** Emadi, F. *et al*. Functionalized Graphene Oxide with Chitosan for Protein Nanocarriers to Protect against Enzymatic Cleavage and Retain Collagenase Activity. *Sci. Rep.* **7**, 42258; doi: 10.1038/srep42258 (2017).

**Publisher's note:** Springer Nature remains neutral with regard to jurisdictional claims in published maps and institutional affiliations.

## Figures and Tables

**Table 1:** Elemental analysis from RBS.

Element→Compound ↓	C%	O%	N%	O/C ratio
CS	48	35	17	0.73
GO	65	35	—	0.54
GO-CS	55	26	19	0.47

**Table 2:** Zeta potentials.

Material	Zeta potential (mV)
GO	−98
CS	+103
GO-CS	+78
BSA	−28
GO-BSA	−102
GO-CS-BSA	+45
Collagenase	−38
GO-collagenase	−103
GO-CS-collagenase	+42

**Table 3:** Loading efficacy.

Percentage →Compound ↓	BSA LE (%)	Collagenase LE (%)
GO	26	35
GO-CS	63	72
